# Bis(piperidin-1-yl)methanone

**DOI:** 10.1107/S1600536811001334

**Published:** 2011-01-15

**Authors:** Richard Betz, Thomas Gerber, Henk Schalekamp

**Affiliations:** a Nelson Mandela Metropolitan University, Summerstrand Campus, Department of Chemistry, University Way, Summerstrand, PO Box 77000, Port Elizabeth 6031, South Africa

## Abstract

The title compound, C_11_H_20_N_2_O, is a urea derivative bearing two piperidine moieties in place of the amino groups. The mol­ecule shows approximate non-crystallographic *C*_2_ symmetry. The six-membered rings adopt ^1^ *C*_4_ and ^4^ *C*_1_ conformations and their mean planes make a dihedral angle of 35.87 (5)°. In the crystal, inter­molecular C—H⋯O contacts connect the mol­ecules into infinite strands along the *a* axis.

## Related literature

For the structures of compounds containing bis(piperidin-1-yl)methanone as a ligand, see: Artali *et al.* (2005 ▶); de Souza *et al.* (2003 ▶). For the graph-set analysis of hydrogen bonds, see: Etter *et al.* (1990 ▶); Bernstein *et al.* (1995 ▶). For puckering analysis, see: Cremer & Pople (1975 ▶).
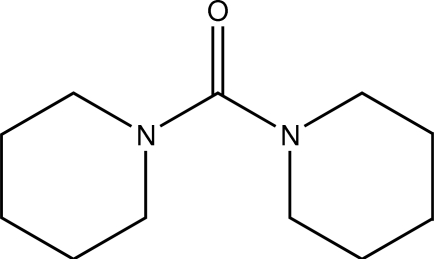


## Experimental

### 

#### Crystal data

C_11_H_20_N_2_O
*M* *_r_* = 196.29
Monoclinic, 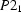

*a* = 6.2193 (2) Å
*b* = 8.8411 (4) Å
*c* = 9.9699 (4) Å
β = 90.791 (1)°
*V* = 548.15 (4) Å^3^
*Z* = 2
Mo *K*α radiation
μ = 0.08 mm^−1^
*T* = 200 K
0.56 × 0.48 × 0.35 mm

#### Data collection

Bruker APEXII CCD diffractometer
9446 measured reflections
1440 independent reflections
1415 reflections with *I* > 2σ(*I*)
*R*_int_ = 0.076

#### Refinement

*R*[*F* ^2^ > 2σ(*F* ^2^)] = 0.030
*wR*(*F* ^2^) = 0.081
*S* = 1.08
1440 reflections
127 parameters
1 restraint
H-atom parameters constrained
Δρ_max_ = 0.23 e Å^−3^
Δρ_min_ = −0.15 e Å^−3^

### 

Data collection: *APEX2* (Bruker, 2010 ▶); cell refinement: *SAINT* (Bruker, 2010 ▶); data reduction: *SAINT*; program(s) used to solve structure: *SHELXS97* (Sheldrick, 2008 ▶); program(s) used to refine structure: *SHELXL97* (Sheldrick, 2008 ▶); molecular graphics: *ORTEPIII* (Farrugia, 1997 ▶) and *Mercury* (Macrae *et al.*, 2006 ▶); software used to prepare material for publication: *SHELXL97* and *PLATON* (Spek, 2009 ▶).

## Supplementary Material

Crystal structure: contains datablocks I, global. DOI: 10.1107/S1600536811001334/jh2250sup1.cif

Structure factors: contains datablocks I. DOI: 10.1107/S1600536811001334/jh2250Isup2.hkl

Additional supplementary materials:  crystallographic information; 3D view; checkCIF report

## Figures and Tables

**Table 1:** Hydrogen-bond geometry (Å, °)

*D*—H⋯*A*	*D*—H	H⋯*A*	*D*⋯*A*	*D*—H⋯*A*
C22—H22*A*⋯O1^i^	0.99	2.50	3.4110 (17)	154

Symmetry code: (i) 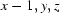.

**Fig. 1.**
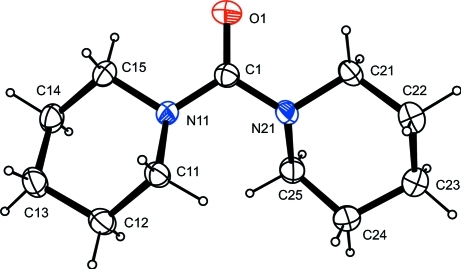
The molecular structure of the title compound, with atom labels and anisotropic displacement ellipsoids (drawn at 50% probability level).

**Fig. 2.**
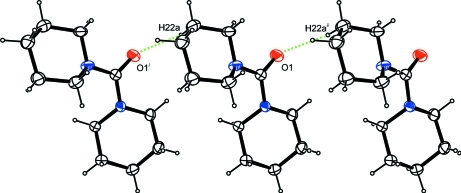
Intermolecular C–H···O contacts, viewed along [0 0 1]. Symmetry operators: i -1 + x, y, z; ii 1 + x, y, z.

**Fig. 3.**
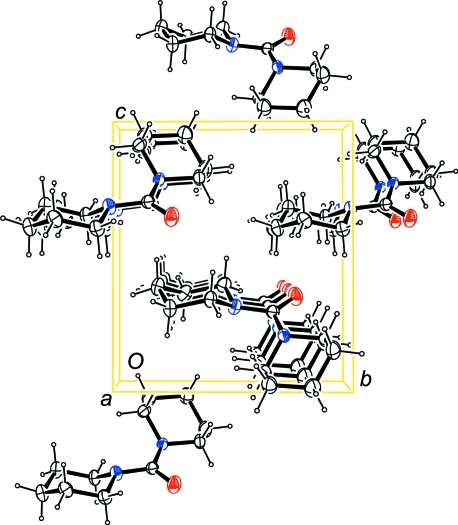
Molecular packing of the title compound, viewed along [-1 0 0] (anisotropic displacement ellipsoids drawn at 50% probability level).

## supplementary crystallographic information

### Comment

Dipiperidin-1-ylmethanone – also known as carbodipiperidid or bis(pentamethylene)urea – is a derivative of urea bearing two piperidine moieties. Given its *N,O* set of donor atoms, it can act as a mono- or a bidentate ligand. Despite this versatility, the coordination chemistry of the title compound has remained nearly unexplored. In a larger study to determine the coordination behaviour of nitrogen- and oxygen-containing ligands, it seemed of interest to determine the structure of the free ligand to enable comparative studies.

The two six-membered rings are present in ^1^*C*_4_ and ^4^*C*_1_ conformation, respectively. The least-square planes defined by their atoms intersect at an angle of 35.87 (5)°. The distance between the two nitrogen atoms was found to be approximately 2.35 Å while both N–O distances were measured around 2.28 Å (Figure 1).

In the crystal structure, C–H···O contacts are observed. If only those contacts are taken into account whose range falls more than 0.2 Å below the sum of van-der-Waals radii of the corresponding atoms, the molecules are connected to infinite strands along the crystallographic *a* axis. The contacts originate from one of the hydrogen atoms in beta-position to the nitrogen atom of the same six-membered ring (Figure 2). In terms of graph-set analysis, the unitary descriptor of this intermolecular interaction is *C*^1^_1_(6).

The molecular packing of the compound is shown in Figure 3.

### Experimental

The structural analysis was done on a single-crystal taken from a commercially obtained (EGA Chemicals) batch of the title compound.

### Refinement

Carbon-bound H-atoms were placed in calculated positions (C—H 0.99 Å) and were included in the refinement in the riding model approximation, with *U*(H) set to 1.2*U*_eq_(C).

### Crystal data

**Table:**

C_11_H_20_N_2_O	*F*(000) = 216
*M**_r_* = 196.29	*D*_x_ = 1.189 Mg m^−^^3^
Monoclinic, *P*2_1_	Mo *K*α radiation, λ = 0.71073 Å
Hall symbol: P 2yb	Cell parameters from 8651 reflections
*a* = 6.2193 (2) Å	θ = 3.1–28.2°
*b* = 8.8411 (4) Å	µ = 0.08 mm^−^^1^
*c* = 9.9699 (4) Å	*T* = 200 K
β = 90.791 (1)°	Platelet, colourless
*V* = 548.15 (4) Å^3^	0.56 × 0.48 × 0.35 mm
*Z* = 2	

### Data collection

**Table:**

Bruker APEXII CCD diffractometer	1415 reflections with *I* > 2σ(*I*)
Radiation source: fine-focus sealed tube	*R*_int_ = 0.076
graphite	θ_max_ = 28.3°, θ_min_ = 3.8°
φ and ω scans	*h* = −8→8
9446 measured reflections	*k* = −11→11
1440 independent reflections	*l* = −13→13

### Refinement

**Table:**

Refinement on *F*^2^	Primary atom site location: structure-invariant direct methods
Least-squares matrix: full	Secondary atom site location: difference Fourier map
*R*[*F*^2^ > 2σ(*F*^2^)] = 0.030	Hydrogen site location: inferred from neighbouring sites
*wR*(*F*^2^) = 0.081	H-atom parameters constrained
*S* = 1.08	*w* = 1/[σ^2^(*F*_o_^2^) + (0.048*P*)^2^ + 0.0376*P*] where *P* = (*F*_o_^2^ + 2*F*_c_^2^)/3
1440 reflections	(Δ/σ)_max_ < 0.001
127 parameters	Δρ_max_ = 0.23 e Å^−^^3^
1 restraint	Δρ_min_ = −0.15 e Å^−^^3^

### Special details

**Table:**

Refinement. Due to the absence of a strong anomalous scatterer, the Flack parameter is meaningless. Thus, Friedel opposites (1259 pairs) have been merged and the item was removed from the CIF.

### Fractional atomic coordinates and isotropic or equivalent isotropic displacement parameters (Å^2^)

**Table:**

	*x*	*y*	*z*	*U*_iso_*/*U*_eq_	
O1	0.75245 (16)	0.25290 (12)	0.64263 (10)	0.0360 (2)	
N11	0.67033 (16)	0.00453 (12)	0.67895 (11)	0.0253 (2)	
N21	0.46865 (18)	0.19864 (11)	0.77644 (10)	0.0267 (2)	
C1	0.63894 (19)	0.15808 (14)	0.69610 (11)	0.0233 (2)	
C11	0.48491 (19)	−0.09425 (14)	0.64910 (13)	0.0262 (2)	
H11A	0.4449	−0.0855	0.5530	0.031*	
H11B	0.3603	−0.0616	0.7026	0.031*	
C12	0.5389 (2)	−0.25768 (15)	0.68181 (14)	0.0304 (3)	
H12A	0.4159	−0.3231	0.6561	0.036*	
H12B	0.5638	−0.2683	0.7796	0.036*	
C13	0.7384 (2)	−0.30892 (16)	0.60767 (15)	0.0320 (3)	
H13A	0.7067	−0.3131	0.5102	0.038*	
H13B	0.7795	−0.4118	0.6378	0.038*	
C14	0.9247 (2)	−0.19995 (16)	0.63433 (14)	0.0308 (3)	
H14A	0.9702	−0.2073	0.7297	0.037*	
H14B	1.0484	−0.2291	0.5785	0.037*	
C15	0.8609 (2)	−0.03795 (15)	0.60269 (13)	0.0283 (3)	
H15A	0.9814	0.0308	0.6260	0.034*	
H15B	0.8298	−0.0278	0.5055	0.034*	
C21	0.4223 (2)	0.36023 (14)	0.78549 (13)	0.0290 (3)	
H21A	0.4544	0.4098	0.6990	0.035*	
H21B	0.5147	0.4067	0.8559	0.035*	
C22	0.1876 (2)	0.38427 (17)	0.81904 (15)	0.0356 (3)	
H22A	0.0962	0.3474	0.7438	0.043*	
H22B	0.1600	0.4938	0.8303	0.043*	
C23	0.1276 (2)	0.30111 (18)	0.94720 (16)	0.0373 (3)	
H23A	−0.0295	0.3086	0.9605	0.045*	
H23B	0.2007	0.3493	1.0250	0.045*	
C24	0.1927 (2)	0.13496 (17)	0.93964 (14)	0.0353 (3)	
H24A	0.1689	0.0862	1.0276	0.042*	
H24B	0.1015	0.0827	0.8719	0.042*	
C25	0.4276 (2)	0.11891 (16)	0.90219 (12)	0.0307 (3)	
H25A	0.5200	0.1613	0.9746	0.037*	
H25B	0.4637	0.0105	0.8920	0.037*	

### Atomic displacement parameters (Å^2^)

**Table:**

	*U*^11^	*U*^22^	*U*^33^	*U*^12^	*U*^13^	*U*^23^
O1	0.0348 (5)	0.0270 (5)	0.0465 (6)	−0.0054 (4)	0.0107 (4)	0.0036 (4)
N11	0.0207 (4)	0.0226 (5)	0.0330 (5)	−0.0014 (4)	0.0068 (4)	−0.0040 (4)
N21	0.0358 (5)	0.0187 (5)	0.0257 (5)	0.0034 (4)	0.0083 (4)	0.0027 (4)
C1	0.0239 (5)	0.0229 (6)	0.0230 (5)	−0.0011 (4)	0.0001 (4)	0.0000 (4)
C11	0.0223 (5)	0.0206 (5)	0.0356 (6)	−0.0014 (4)	0.0006 (4)	−0.0020 (4)
C12	0.0275 (6)	0.0220 (6)	0.0417 (7)	−0.0002 (5)	0.0015 (5)	0.0005 (5)
C13	0.0313 (6)	0.0232 (6)	0.0416 (7)	0.0034 (5)	0.0002 (5)	−0.0052 (5)
C14	0.0242 (5)	0.0317 (7)	0.0366 (6)	0.0040 (5)	0.0029 (4)	−0.0058 (5)
C15	0.0238 (5)	0.0284 (6)	0.0329 (6)	−0.0002 (5)	0.0082 (4)	−0.0035 (5)
C21	0.0364 (6)	0.0178 (5)	0.0329 (6)	0.0005 (5)	0.0061 (5)	−0.0008 (5)
C22	0.0373 (7)	0.0259 (6)	0.0437 (7)	0.0059 (5)	0.0037 (5)	0.0013 (5)
C23	0.0359 (7)	0.0322 (7)	0.0442 (7)	0.0032 (6)	0.0129 (5)	−0.0007 (6)
C24	0.0391 (7)	0.0287 (7)	0.0386 (7)	−0.0006 (6)	0.0142 (5)	0.0029 (6)
C25	0.0394 (7)	0.0266 (6)	0.0263 (5)	0.0053 (5)	0.0077 (4)	0.0055 (5)

### Geometric parameters (Å, °)

**Table:**

O1—C1	1.2229 (16)	C14—H14B	0.9900
N11—C1	1.3825 (15)	C15—H15A	0.9900
N11—C15	1.4661 (15)	C15—H15B	0.9900
N11—C11	1.4736 (15)	C21—C22	1.5167 (19)
N21—C1	1.3839 (15)	C21—H21A	0.9900
N21—C21	1.4604 (16)	C21—H21B	0.9900
N21—C25	1.4639 (15)	C22—C23	1.525 (2)
C11—C12	1.5178 (18)	C22—H22A	0.9900
C11—H11A	0.9900	C22—H22B	0.9900
C11—H11B	0.9900	C23—C24	1.526 (2)
C12—C13	1.5222 (18)	C23—H23A	0.9900
C12—H12A	0.9900	C23—H23B	0.9900
C12—H12B	0.9900	C24—C25	1.5193 (19)
C13—C14	1.5273 (19)	C24—H24A	0.9900
C13—H13A	0.9900	C24—H24B	0.9900
C13—H13B	0.9900	C25—H25A	0.9900
C14—C15	1.5182 (19)	C25—H25B	0.9900
C14—H14A	0.9900		
			
C1—N11—C15	115.61 (10)	C14—C15—H15A	109.6
C1—N11—C11	119.70 (10)	N11—C15—H15B	109.6
C15—N11—C11	112.30 (10)	C14—C15—H15B	109.6
C1—N21—C21	116.26 (10)	H15A—C15—H15B	108.1
C1—N21—C25	121.00 (10)	N21—C21—C22	110.00 (11)
C21—N21—C25	112.41 (10)	N21—C21—H21A	109.7
O1—C1—N11	122.40 (12)	C22—C21—H21A	109.7
O1—C1—N21	121.71 (12)	N21—C21—H21B	109.7
N11—C1—N21	115.88 (10)	C22—C21—H21B	109.7
N11—C11—C12	110.53 (10)	H21A—C21—H21B	108.2
N11—C11—H11A	109.5	C21—C22—C23	111.39 (12)
C12—C11—H11A	109.5	C21—C22—H22A	109.3
N11—C11—H11B	109.5	C23—C22—H22A	109.3
C12—C11—H11B	109.5	C21—C22—H22B	109.3
H11A—C11—H11B	108.1	C23—C22—H22B	109.3
C11—C12—C13	111.00 (11)	H22A—C22—H22B	108.0
C11—C12—H12A	109.4	C22—C23—C24	110.76 (12)
C13—C12—H12A	109.4	C22—C23—H23A	109.5
C11—C12—H12B	109.4	C24—C23—H23A	109.5
C13—C12—H12B	109.4	C22—C23—H23B	109.5
H12A—C12—H12B	108.0	C24—C23—H23B	109.5
C12—C13—C14	110.45 (10)	H23A—C23—H23B	108.1
C12—C13—H13A	109.6	C25—C24—C23	111.02 (11)
C14—C13—H13A	109.6	C25—C24—H24A	109.4
C12—C13—H13B	109.6	C23—C24—H24A	109.4
C14—C13—H13B	109.6	C25—C24—H24B	109.4
H13A—C13—H13B	108.1	C23—C24—H24B	109.4
C15—C14—C13	111.32 (10)	H24A—C24—H24B	108.0
C15—C14—H14A	109.4	N21—C25—C24	110.20 (11)
C13—C14—H14A	109.4	N21—C25—H25A	109.6
C15—C14—H14B	109.4	C24—C25—H25A	109.6
C13—C14—H14B	109.4	N21—C25—H25B	109.6
H14A—C14—H14B	108.0	C24—C25—H25B	109.6
N11—C15—C14	110.17 (10)	H25A—C25—H25B	108.1
N11—C15—H15A	109.6		
			
C15—N11—C1—O1	5.50 (17)	C12—C13—C14—C15	−53.49 (14)
C11—N11—C1—O1	−133.79 (13)	C1—N11—C15—C14	158.70 (10)
C15—N11—C1—N21	−175.32 (10)	C11—N11—C15—C14	−59.06 (13)
C11—N11—C1—N21	45.39 (15)	C13—C14—C15—N11	55.89 (14)
C21—N21—C1—O1	4.47 (18)	C1—N21—C21—C22	154.25 (11)
C25—N21—C1—O1	−137.93 (13)	C25—N21—C21—C22	−60.21 (15)
C21—N21—C1—N11	−174.72 (11)	N21—C21—C22—C23	55.64 (16)
C25—N21—C1—N11	42.88 (16)	C21—C22—C23—C24	−52.35 (17)
C1—N11—C11—C12	−160.27 (11)	C22—C23—C24—C25	52.16 (17)
C15—N11—C11—C12	59.21 (14)	C1—N21—C25—C24	−155.95 (11)
N11—C11—C12—C13	−55.74 (14)	C21—N21—C25—C24	60.33 (15)
C11—C12—C13—C14	53.25 (14)	C23—C24—C25—N21	−55.55 (16)

### Hydrogen-bond geometry (Å, °)

**Table:**

*D*—H···*A*	*D*—H	H···*A*	*D*···*A*	*D*—H···*A*
C22—H22A···O1^i^	0.99	2.50	3.4110 (17)	154

Symmetry codes: (i) *x*−1, *y*, *z*.
